# 

**Published:** 1884-01

**Authors:** 


| | PAGE |
|---|---|
| ORIGINAL COMMUNICATIONS.—Scarlet Fever in Horses.—Dr. John C. Peters | 1 |
| Some diseases of the Ostrich. W. A. Conklin, D. V. S. | 14 |
| The ancients on Equine Age Marks. William H. Clarke | 19 |
| The early history of Veterinary Medicine in the United States. R. Jennings, V.S. | 25 |
| The Ophthalmoscopic appearance of the Fundus of the Eye of the Horse. Prof. D. Berlin, Stuttgard | 32 |
| History of Tuberculosis, with a special consideration of Tuberculosis in Cattle. Dr. Albert Johne | 44 |
| Comparative Physiology of Menstruation. Alfred Wiltshire, M.D., F.R.C.P., London | 58 |
| EDITORIAL.—The Scientific method in Veterinary Education—The International Veterinary Congress—To the Veterinary Profession in the United States.—An Epidemic of Trichiniasis on the Jordan—Sanitation and Infectious Diseases—Gratuitous treatment at Clinics—The New York State Convention—Experiments with reference to the Development and Regeneration of Tendons—Annual Report of the Royal Veterinary Institute, Berlin, Prussia, for the year 1882-'3—Seventh Annual Report of the Royal Commission—How slaughtered animals are inspected at the Abattoir at Berlin, Germany—A contribution to the Experimental Diagnosis of Glanders—Eulenberg—A case of Irido-Choroiditis—Columbia Veterinary College, Opening of the Sixth Session—Prof. Dick's Statue | 66-94 |
| CASE DEPARTMENT.—A Singular Case of Congenital Misplacements in a Dog—Was the Mare Pregnant?—Emesis in a Horse—Rupture of the Sclerotica in a Horse—Cholesterin Crystals in the Vitreous Humour of the Eye in a Horse—Persistent Arteria Hyaloidea or Filaria in the Vitreous Humour of the Eye of the Horse | 95-100 |
| CORRESPONDENCE | 101 |
| REVIEWS | 107 |
| PROGRESS OF VETERINARY SCIENCE.—Officers of American Public Health Association—Hog Cholera—Texas Cattle Fever—Actinomycosis discovered in American Cattle—Propagation of Diphtheria by Chickens—The Contagion of Tuberculosis—Diarrhœa in a Camel—Pneumocele in a Cow—Acute Gastro-Enteritis in a Bear—Medicine as practised by animals Posology and Therapeutics—Inoculation with Virus of Farcy Glanders—Syphilitic Inoculation of Monkeys—Bone Disease in Monkeys | 110-115 |
| NEWS AND MISCELLANY.—Tuberculosis in wild animals—Prolonged Lactation in a Cow—Heroic Treatment—Rheumatism in Cattle—Shoulder Lameness | 116 |

